# A digital media literacy intervention for older adults improves resilience to fake news

**DOI:** 10.1038/s41598-022-08437-0

**Published:** 2022-04-09

**Authors:** Ryan C. Moore, Jeffrey T. Hancock

**Affiliations:** Department of Communication, Stanford University, Stanford, CA USA

**Keywords:** Psychology, Human behaviour

## Abstract

Older adults are especially susceptible to fake news online, possibly because they are less digitally literate compared to younger individuals. Interventions for older adults have emerged to improve digital literacy, although there has been little evaluation of their effectiveness in improving older adults’ resilience to fake news. We report the results of a digital literacy intervention for older adults administered during the 2020 U.S. election. The intervention was a 1-hour, self-directed series of interactive modules designed to teach concepts and skills for identifying misinformation online. Consistent with our pre-registered hypothesis, older adults (*M*_age_ = 67) in the treatment condition (N = 143) significantly improved their likelihood of accurately discerning fake from true news from 64% pre-intervention to 85% post-intervention. In contrast, older adults in the control condition (N = 238) did not significantly improve (from 55% to 57%). The treated older adults were also more likely to employ strategies for identifying misinformation online compared to pre-intervention and the control group.

## Introduction

Recent research has identified older adults as a demographic group especially susceptible to fake news online. For example, during the 2016 U.S. presidential campaign, people 65 and older were twice as likely to be exposed to fake news on Twitter and seven times more likely to share fake news on Facebook than 18–29 year olds^1,2^. Analyzing across a nationally representative sample’s mobile, desktop, and television media consumption for a nearly 3 year period, Allen et al. found that older individuals were substantially greater consumers of fake news than younger people^3^.

Scholars have postulated that older adults’ limited digital literacy may explain their heightened susceptibility to fake news online^4^. Here, we use “digital literacy” to refer to what researchers have also called digital information literacy, or the ability to analyze and evaluate information encountered online, including judgment of information reliability or evaluation of sources and evidence^5,6^. In general, research has shown that older adults possess lower levels of digital and internet-related skills relative to younger individuals^7–9^. Several factors likely contribute to this pattern. For instance, unlike younger individuals, older adults are not “digital natives” and may have less experience using contemporary media technologies and platforms as they were not as large a part of their professional and personal lives^10^. Further, technologies themselves are often designed in such a way that are more difficult for older adults to use (e.g., small text sizes, reliance on touchscreen inputs)^11^.

With these digital challenges in mind, organizations have begun developing digital literacy training programs for older adults to aid them in sorting fact from fiction online, although to date there has been little formal evaluation of such interventions. In this paper, we report results from our collaboration with MediaWise, a non-profit journalism organization, who administered a digital literacy training program for older adults, *MediaWise for Seniors*, during the 2020 presidential election in the United States^12^. This intervention was a 1-h online course which, through the use of text, videos, interactive exercises, and short quizzes, taught skills important for verifying the credibility of information encountered online, such as “lateral reading” (opening new web browser tabs and conducting searches to see what other sources say about a claim) and reverse image searching^13^. Relative to other digital literacy training resources, *MediaWise for Seniors* was designed specifically for older adults—the training progressed at a slower pace, employed trusted instructors that would be familiar to the population (e.g., Joan Lunden, Christiane Amanpour), and focused heavily on Facebook since older adults are significantly more likely to use Facebook than other social media platforms and are currently Facebook’s fastest growing user group^14^.

By administering surveys to older adults who took MediaWise’s training before and after the training, in addition to administering the same surveys to a control group of older adults who did not take the training at the same points in time, we were able to examine the effect of participation in the digital literacy training on three key outcomes: (1) Older adults’ ability to correctly identify true and false news headlines, (2) their likelihood of doing research online in order to inform their judgments about the veracity of those headlines, and (3) their comprehension of skills and techniques important for identifying misinformation online (e.g., lateral reading, reverse image searching).

## Possible intervention outcomes

### Old dogs, new clicks?

Although digital media literacy training has been shown to boost younger individuals’ abilities to identify online misinformation^15,16^, it is not yet clear whether such programming can be similarly effective for older adults. While there are efforts to create training and products to improve the digital media literacy of older adults, few interventions exist relative to those designed for younger or more general audiences, and even fewer have been rigorously evaluated.

One possible outcome is that digital literacy training for older adults has no effect on their ability to detect misinformation online. Indeed, recent evidence from a large-scale digital media literacy intervention in India (not focused specifically on older adults) supports this possibility^17^. For older adults in particular, given their documented struggles with digital literacy skills, it may be difficult to successfully confer new skills and techniques, particularly in a short period of time. Furthermore, previous work has shown that older adults often feel skeptical of new technologies or perceive that they’ll be difficult to use^18,19^. This skepticism and anxiety could serve as barriers to new learning for digital media literacy.

### Undermining trust in news

A more pernicious possible outcome is that digital literacy training could make individuals less trusting of all news, both false *and* truthful news. To understand how this might happen, we can look to the literature on deception detection in interpersonal communication. For example, Levine’s *Truth-Default Theory*^20^ argues that people interpret incoming communication from other people as truthful by default. This tendency is referred to as the *truth bias*. As a consequence, in deception detection tasks in which people are asked to judge the veracity of messages as true or false, a pattern of results persists across nearly all published studies in which people are more accurate at judging the veracity of true messages than false messages, simply because they are more likely in general to interpret messages as true. This empirical trend is known as the *veracity effect* and it persists because people are truth biased.

It is possible, however, for people to become less truth biased under certain conditions. For example, experimentally priming people to be suspicious of message senders decreased the extent to which they rated messages from those senders to be true^21,22^. As a consequence, these suspicious participants became less accurate at detecting true messages but more accurate at detecting false messages. A digital media literacy intervention could have a similar effect by making people more suspicious of news overall. By providing people with examples of information online that turned out to be false and by priming them to think more critically about the veracity of online information, they may become more suspicious of the media they encounter online after the intervention. Indeed, recent evidence indicates that when evaluating news, people tend to be less truth biased compared to other types of information^23^, suggesting that there may already be a significant amount of suspicion when it comes to the veracity of news content. This could be due to a variety of factors, including declining trust in institutions^24^ as well as significant media coverage of misinformation generating heightened suspicion toward news^25^.

If digital media literacy interventions indeed make individuals more suspicious of news overall, we would expect them to become more accurate at detecting false news but less accurate at detecting true news after participating in an intervention. Indeed, an evaluation of a recent digital literacy intervention in the U.S. and India by Guess et al. provides support for this possibility. Individuals who participated in their digital literacy intervention not only became more likely to classify false news as false post-intervention but also became more likely to mistakenly classify true news as false^26^.

Ideally, interventions will make participants more accurate at judging the veracity of both false *and* true news. We refer to this as the ability to engage in *discriminant trust*. Understanding whether interventions improve people’s ability to engage in discriminant trust of news or instead cause them to become more suspicious of news overall, making them more accurate at judging the veracity of false news but less accurate at judging true news, is important. Recent work indicates that the vast majority of news in individuals’ everyday media diets is not false (approximately 99% according to Allen et al.^3^). To clarify, we mean that a small amount of news in people’s media diets is that which has been fact-checked/verified to be false. However, most news that people encounter in their daily lives has likely not been fact checked and a great deal may lie somewhere between true and false (e.g., truthful news presented alongside a misleading or unsubstantiated implication). We recognize that this is a limitation of research estimating the prevalence of true and false news in individuals’ media diets, which often relies on lists or databases of fact-checked or verified false news^1–3,27^. That said, this work finds that false news comprises a far smaller percentage of people’s media diets than true news.

If digital media literacy interventions make individuals more suspicious of all news, this may mean that they become less accurate at judging the veracity of the majority of information they consume on a daily basis, while becoming more accurate at judging false news, which makes up a comparatively small amount. To examine which of these possibilities results from digital literacy interventions, it is critical to examine changes in individuals’ detection abilities for true and false news separately, in addition to examining their detection abilities overall.

### Bolstering resilience to fake news and trust in real news

A third possibility is that digital literacy training will successfully build resilience to online misinformation among older adults while also enhancing their trust in real news. There is some limited anecdotal evidence that small-scale trainings for older adults designed to help with developing digital skills to identify misinformation online have been successful^28^. Additionally, despite the fact that older adults have struggled with digital literacy in the past more so than younger individuals, research in human–computer interaction suggests that due to improvements in software and hardware design^29^, as well as a boom in older adults’ adoption of modern digital technologies^30,31^, older adults may be well-positioned to improve their digital and online abilities now relative to the past. For these reasons, in addition to *MediaWise for Seniors*’ tailoring of their content for older adults, we expected that the training would be effective in improving participants’ ability to correctly identify true and false news headlines. More specifically, we preregistered the following hypothesis: among those who participate in *MediaWise for Seniors*, the accuracy with which individuals classify news headlines as true or false will be greater after the course compared to before.

However, as was discussed, while overall accuracy is informative, an intervention should improve individuals’ *discriminant trust* in news—that is, their ability to accurately judge true news as true and false news as false. Thus, in addition to examining the effect of *MediaWise for Seniors* on overall headline veracity judgment accuracy, we also examine its effect on true and false headlines separately to see whether or not digital literacy training can help individuals build discriminant trust.

In addition to people’s ability to accurately judge the veracity of news headlines, does participating in digital literacy training actually improve people’s comprehension of skills and techniques important for identifying misinformation online? Further, will older adults *use* those strategies at greater rates after taking the course in order to verify the veracity of online information?

## Methods

### Intervention details

The MediaWise for Seniors intervention was a self-directed online course which taught digital media literacy skills and techniques helpful for verifying the credibility of information online, such as lateral reading and reverse image searching (see Supplementary Materials for more information about all skills covered in the course). These skills and techniques were selected based on digital literacy tools and techniques discussed in prior work to be effective at improving people’s ability to identify credible information (e.g., lateral reading^13^; reverse image search^32^), however we note that the efficacy of teaching these skills to an older adult population, to our knowledge, has not been formally evaluated prior to our study.

The course was designed to take approximately 1 h to complete and participants navigated through the sections of the course at their own pace. Participants in MediaWise for Seniors could complete the course on any device with a web browser and participants had to login to MediaWise’s website to access the course, so they were able to complete part of it, take a break, and return later to pick up where they left off. The contents of the course were highly multimodal—information was presented as text, in photos and infographics, through instructional videos, and through interactive examples where participants were walked through examples of encountering online misinformation. Researchers interested in replicating our study can contact the authors for access to the full intervention.

### Participants and procedure

From September 24 to December 2, 2020, 143 older adults recruited by MediaWise completed the *MediaWise for Seniors* course and both our pre and post-course surveys (*M*_age_ = 67.2 years, 67.4% female, 86.8% white). From October 1 to November 6, 2020, 238 older adults recruited from online survey purveyor Lucid also completed both our pre and post surveys, without taking MediaWise’s course or being exposed to any other control stimuli (*M*_age_ = 63.8 years, 60.5% female, 89.5% white). The Lucid respondents served as a control group to compare to our course enrollee group. The control group took the same pre- and post-course surveys but they did *not* participate in the *MediaWise for Seniors* course. The study was approved by the Stanford University Institutional Review Board (Protocol # IRB-58508). Informed consent was obtained from all participants and the study protocol was performed in accordance with relevant guidelines and regulations. Our pre-registration document can be found at: https://osf.io/jv8hk?view_only=51e12172bc8542ee86211d58ae150bdb.

### Measures

Of primary interest in our surveys was a deception detection task for news headlines. In both the pre and post surveys, individuals were asked to rate the veracity of 6 headlines from 1 (definitely false) to 7 (definitely true), for a total of 12 headline judgments across the two surveys. Each survey contained 3 true and 3 false news headlines, and the 6 headlines in the pre-survey were different from the 6 headlines in the post-survey (see Table S1). The choice of whether to test participants in studies of misinformation interventions on the same or different news items in pre and post-intervention surveys can be a consequential research design choice^33^. For example, if an intervention is being evaluated without a control group, using different news items in the pre and post-intervention surveys may introduce a confound for measuring the effect of the intervention if the news items in the post-intervention survey are easier to detect as true or false than the news items in the pre-intervention survey. In this study, the use of a control group alleviates this concern.

Per survey, one true (false) headline was pro-Republican, meaning that it was congenial with a stereotypical Republican point of view, 1 true (false) headline was pro-Democrat, and 1 true (false) headline was comparatively non-partisan in nature (see Table S1). All 12 headlines were taken from articles or posts fact-checked by professional fact-checking organization Snopes. After providing veracity judgments for the headlines in each survey, respondents were asked if they did any research online to inform their judgment of the veracity of each headline (yes/no). We recognize that the phrase “doing one’s research” may mean different things to different people and may be politically charged in the current information environment^34^. For each headline, participants were asked if they did research on that headline via the following language: “Did you do any research on this headline before you provided a judgment as to how likely you believed it to be true? For example, opening up a new tab to search for information about the story, copy-and-pasting the headline into a search engine, etc.”.

In addition, we measured levels of skill on the concepts and skills taught in the course important for being able to identify disinformation online. We adopted Hargittai and Hsieh’s widely-used measure of internet skills to measure respondents’ level of skill for these techniques and concepts^35^, in which individuals are asked to rate their understanding of internet-related terms (e.g., firewall) from 1 (no understanding) to 5 (full understanding). We replaced Hargittai and Hsieh’s terms with 6 skills and concepts taught in *MediaWise for Seniors* and important for detecting disinformation online, including lateral reading, click restraint, reverse image searching, Wikipedia page features, search engine query optimization, and search engine filters^5,13^ (for more information about these skills see the Supplementary Materials).

## Results

### News headline classification task

First, we examine our pre-registered hypothesis: participants in the *MediaWise for Seniors* intervention will classify news headlines as true or false more accurately after the course compared to before (data and code to reproduce results available upon request). To evaluate individuals’ ability to accurately judge the veracity of news headlines, we estimated logistic regression models in which the dependent variable was a binary variable indicating if a respondents’ veracity judgment for a particular headline was correct or incorrect. To create that variable, we dichotomized respondents’ 7-point veracity judgments such that, for true headlines, ratings of 5–7 were coded “1” for correct and ratings of 1–3 were coded “0” for incorrect. For false headlines, ratings of 1–3 were coded “1” while ratings of 5–7 were coded “0”. Neutral ratings of 4 were excluded from analysis^36^. The independent variables included a binary indicator variable for whether judgments came from the intervention group (i.e., those who participated in *MediaWise for Seniors*) (1) or from the control group (0), a binary indicator of whether judgments came from the post-intervention survey (1) or pre-intervention survey (0), and our key variable of interest was the interaction of those two variables. Standard errors were clustered on participants to account for repeated observations. All statistical analyses were performed using R^37^ and figures were made using Stata^38^.

**Figure 1:**
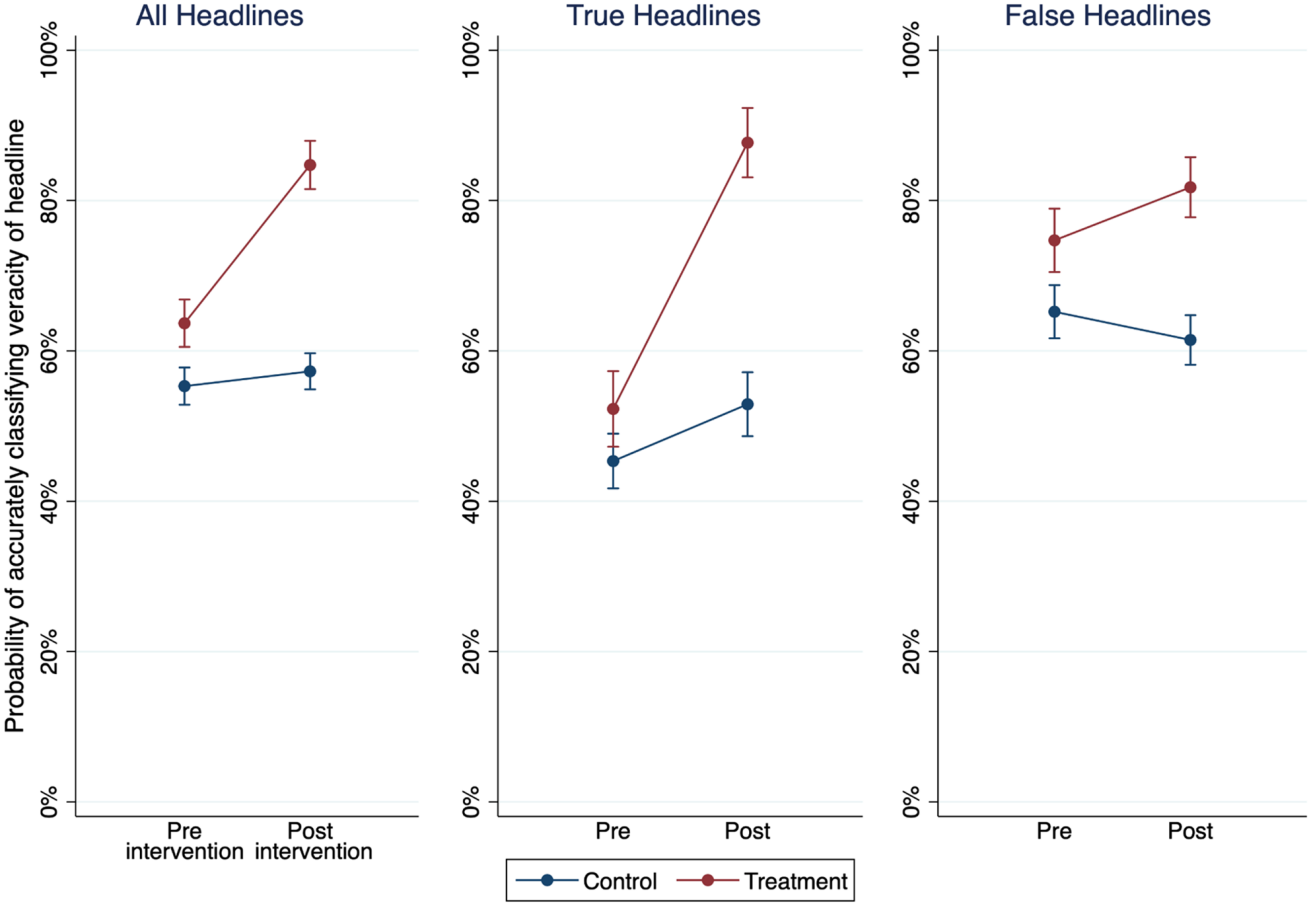
Predicted probabilities of correctly judging news headline veracity. Note: Predicted probability of accurately judging the veracity of news headlines broken down by all news headlines, among only true news headlines, and among only false news headlines (from models in Table S2). Higher values indicate greater probability of correctly judging the veracity of a given headline. Blue points represent the control group and red points represent the Intervention group (i.e., those who took *MediaWise for Seniors*). Bars are 95% confidence intervals. This figure was made using Stata^38^.

As can be seen in Table S2, we obtained a positive and significant interaction between the course enrollee and post-intervention indicator variables for all news headlines (i.e., true and false news headlines combined; B = 1.073, *SE* = 0.159, *p* < 0.001). This interaction suggests that improvement in the ability to accurately judge the veracity of news headlines among the intervention group from pre-intervention to post-intervention was significantly greater than change in that ability among the control group. To interpret this interaction, we created predicted probability plots (Fig. 1). The intervention group rose from a pre-intervention probability of accurately judging the veracity of a headline of 64% [95% CI 61–67%] to a post-intervention probability of 85% [95% CI 82–88%] (Fig. 1, All Headlines). The control group did not experience a significant increase in accuracy (pre-intervention probability = 55% [95% CI 53–58%], post-intervention probability = 57% [95% CI 55–60%]). This pattern provides support for our hypothesis.

To examine if those who participate in *MediaWise for Seniors* display an increase in *discriminant trust* after the course compared to before, we can perform a similar analysis as that testing our pre-registered hypothesis. However, instead of the dependent variable containing veracity judgments for both true and false headlines, we estimate separate models for true news and for false news. As can be seen in Table S2, we obtained a positive and significant interaction between the Intervention Group and Post-intervention variables in both the models for true headlines (Model 2; B = 1.570, *SE* = 0.265, *p* < 0.001) and false headlines (Model 3; B = 0.580, *SE* = 0.120, *p* < 0.01). The intervention group rose from a pre-intervention probability of accurately judging the veracity of a true headline of 52% [95% CI 47–57%] to a post-intervention probability of 88% [95% CI 83–92%] (Fig. 1, True Headlines) and from a post-intervention probability of accurately judging the veracity of a false headline of 75% [95% CI 70–79%] to a post-intervention probability of 82% [95% CI 78–86%] (Fig. 1, False Headlines). Taken together, these models demonstrate that those who participated in *MediaWise for Seniors* improved their ability to accurately classify both false *and* true news stories after taking the course, demonstrating that this digital literacy training improved discriminant trust. Substantively similar results to those presented in Table S2 are obtained if we use non-dichotomized headline veracity judgments (see Table S3). See also the Supplementary Materials for additional analyses of the headline classification task results (by headline [Figure S1] and by congenial vs. non-congenial headlines [Table S7]).

### Comprehension and use of digital media literacy techniques

**Figure 2:**
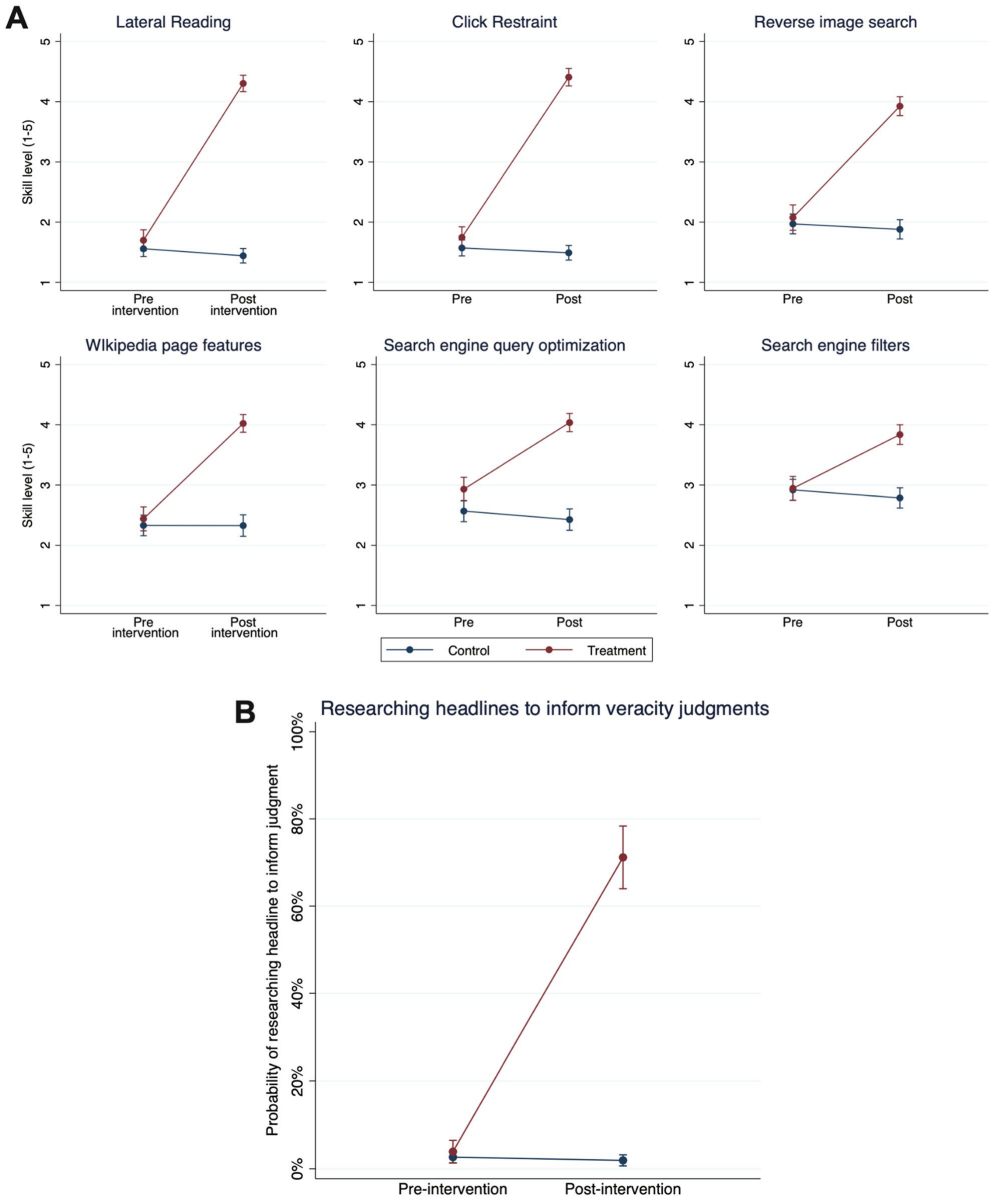
Predicted values of skill levels and probability of researching headlines. Note: Panel (**A**): Predicted values of skill level on six digital literacy skills taught in MediaWise for Seniors important for identifying online misinformation (Table S4). Individuals rated their own skill level on each skill from 1 to 5 where a rating of 1 represents no understanding of that skill and a rating of 5 represents full understanding. Higher values indicate greater probability of correctly judging the veracity of a given headline. Blue points represent the control group and red points represent the intervention group (i.e., those who took MediaWise for Seniors). Bars are 95% confidence intervals. Panel (**B**): Predicted probability of reporting doing research on a headline to inform one’s judgment of its veracity (Table S4). Higher values indicate greater probability of correctly doing research on a headline. Bars are 95% confidence intervals. This figure was made using Stata^38^.

In addition to their performance on the headline classification task, we examine whether those who participate in *MediaWise for Seniors* report greater understanding or use of skills and techniques important for identifying misinformation online after the course compared to before. To explore understanding of skills and techniques, we estimated 6 linear regression models with the same independent variables as those in the headline task models. The dependent variable in each model was participants’ ratings of their skill level (1–5) on one of the 6 digital skills important for identifying misinformation taught in the course. Standard errors were clustered on participants. For all 6 models, we obtained a positive and significant interaction between the Intervention Group and Post-intervention variables, indicating that the increase in understanding of digital literacy skills among the intervention group from pre-intervention to post-intervention was significantly greater than change in understanding among the control group (see Table S4). As can be seen in Fig. 2A, for all six skills, the intervention group experienced significant increases in skill level from pre to post-intervention, while the control group’s skill levels stayed constant (all 95% CIs for control group pre to post skill levels overlap).

To see whether the *use* of techniques increased as a result of taking the course, we can examine the reported rates of researching the headlines in the survey in order to inform their veracity judgments. If individuals report using this strategy, they are engaging in lateral reading, a skill education researchers have demonstrated is important for identifying online misinformation^13^. To examine this, we estimated a logistic regression model in which the independent variables were the same as the previously described models and the dependent variable was a binary variable indicating whether, for a given headline, participants reported doing research to inform their judgment of that headline’s veracity (1) or did not do research to inform their judgment (0). Standard errors were clustered on participants. We obtained a positive and significant interaction between the Intervention Group and Post-intervention variables (B = 4.436, *SE* = 0.505, *p* < 0.001), indicating that the increase in the likelihood of reporting doing research to inform a headline veracity judgment among the intervention group from pre-intervention to post-intervention was significantly greater than change in the likelihood of doing research among the control group (see Table S4).

To interpret this interaction further, we plotted the predicted probability of doing research for both the intervention and control groups in the pre- and post-intervention surveys (Fig. 2B). The intervention group rose from a pre-intervention probability of doing research to inform their headline judgments of 4% [95% CI 1–6%] to a post-intervention probability of 71% [95% CI 64–78%]. Because we do not observe this pattern in the control group (their pre-intervention researching probability = 3% [95% CI 1–4%], post-intervention probability = 2% [95% CI 1–3%]), it is unlikely this effect in the MediaWise enrollee group is driven by priming in the survey instrument. Taken together, these findings demonstrate that those who took *MediaWise for Seniors* showed significantly greater understanding *and* use of skills and techniques important for identifying misinformation online after the course compared to before.

### Addressing self-selection

One possibility that may explain our promising results is that individuals in our treatment group, who self-selected into participating in *MediaWise for Seniors* and electing to take our surveys, may have had a greater motivation to improve their digital literacy relative to other older adults who would not elect to participate in such an intervention. In an attempt to account for this, we included a question in the control group’s pre-intervention survey which displayed marketing materials for the MediaWise for Seniors course and asked each control group respondent if they would be interested in participating in the course. Analyzing the intervention group compared to the subset of the control group who indicated that they would be willing to participate in *MediaWise for Seniors* (N = 80), we obtain results substantively similar to those described above (see Tables S5 and S6). That is, even when comparing the intervention group to those in the control group who expressed interest in participating in the intervention, the intervention group still experienced a significant pre to post course increase in the likelihood of accurately judging the veracity of both true and false news headlines as well as significantly greater understanding *and* use of skills and techniques important for identifying misinformation online.

## Discussion

The older adults who took the *MediaWise for Seniors* intervention showed an improved ability to accurately classify true *and* false news after taking the course, displayed greater comprehension of several skills important for identifying misinformation online, and were more likely to report doing research on news stories before making judgments about their veracity. None of these changes were observed in a control group of older adults. This paper represents, to our knowledge, the first systematic analysis of a digital literacy intervention aimed at helping older adults identify misinformation online. The results reported here indicate that digital literacy interventions are a viable strategy to assist older adults at improving their ability to sort fact from fiction online, important given growing evidence that they are often targeted by disinformation campaigns and other malicious actors online^39^.

Contrary to some prior digital media literacy interventions^26^, the *MediaWise for Seniors* intervention appears to have improved individuals’ abilities to accurately detect both true and false news, what we refer to as the ability to engage in *discriminant trust*: trusting reliable news while distrusting false news. Significantly improving the likelihood that individuals judge true news as true and false news as false is an ideal outcome for an intervention like this. An important question, however, is whether other intervention outcomes, such as participants becoming more likely to judge false news as false but also true news as false, are also positive outcomes.

On the one hand, given that most of the news individuals encounter in daily life is not false^3^, it may be undesirable to make people more accurate at judging the accuracy of content that constitutes a small fraction of their news diet (false news) at the expense of content that makes up a substantially larger portion (true news). On the other hand, the impact of inaccurately assessing the veracity of fake news may be outsized (e.g., contributing to radicalization, polarization, violence), thereby justifying the trade off of making people more accurate at detecting fake news with becoming less accurate at detecting true news. Furthermore, the research which has established that false news comprises a very small percentage of the news which individuals are exposed to operationalizes “false news” as that which has been fact-checked or verified to be false^1–3,27^. News which has not necessarily been fact-checked to be false can still be misleading or polarizing, and increasing people’s suspicion of those pieces of news may be desirable. More work is needed to think through these trade offs, but it does appear that *MediaWise for Seniors* built discriminant trust in this study, at least among news which was fact-checked to be true or false.

Relatedly, our results indicate that, at baseline, individuals were more likely to accurately detect false news rather than true news (see Fig. 1). This is the opposite pattern of results that would be predicted by the *Truth-Default Theory*, which states that because people are truth-biased (i.e., they interpret most messages as true), they are usually more likely to accurately detect true than false messages (an empirical phenomena known as the veracity effect)^20^. While the Truth-Default Theory was developed in the context of interpersonal communication, our work joins others who have found that individuals may not be truth-biased in the domain of news^23^. To our knowledge, however, this is the first time this effect has been documented among older adults. This finding has implications for both the contents of misinformation interventions and the measurement of their efficacy. Content-wise, our results clearly emphasize a need to make the point explicit in interventions like *MediaWise for Seniors* that individuals should not assume all incoming news is false but rather emphasize the conditions under which people should be more or less suspicious of the veracity of news. Measurement-wise, if it is the case that individuals are significantly more likely to accurately detect false compared to true news at baseline, then it may be more difficult to detect improvements to people’s ability to identify false news compared to improvements in people’s ability to identify true news. Indeed, this may help explain the difference between the 36% increase pre to post intervention in our study’s treatment group’s ability to detect true news and the 7% increase in their ability to detect false news. Further documenting the potential absence of a truth-bias in the news domain and understanding how it may impact the efficacy and design of interventions will be important for future work to consider.

While our results are encouraging, our study should be interpreted in light of its limitations. First, although our study was high in external validity (i.e., we studied a real intervention as it was delivered in the field), its internal validity could be improved. Those who took *MediaWise for Seniors* and participated in our surveys self-selected into doing so, and likely have a greater motivation to improve their digital literacy relative to other older adults who would not elect to participate in such an intervention. As was presented in the “Results” section, it does appear that our intervention participants still experienced significant post-intervention improvements in accuracy and digital literacy relative to a subset of the control group who were presented marketing materials for *MediaWise for Seniors* and indicated that they would be interested in participating in it. Still, future work should study these types of interventions using an experimental framework, in which individuals are randomly assigned to an intervention to improve causal inferences that can be drawn about digital literacy interventions’ effects. In addition, we were not able to capture detailed data on engagement with the intervention for this study of *MediaWise for Seniors* (e.g., total time spent on the course, number of logins to the course), but future work should capture engagement metrics when possible to explore how differences in engagement may lead to differences in efficacy among those exposed to a digital literacy intervention.

Additionally, while our study can speak to the effects of *MediaWise for Seniors* as a whole, it is limited in its ability to isolate exactly what about or contained within the intervention made it effective. Indeed, MediaWise for Seniors was a complex intervention which covered a variety of different digital literacy skills and techniques in a variety of modalities (text, photos, videos, interactive exercises) in a way that was tailored to the older adult population and which lasted a particular amount of time (approximately 1 h). Any one of these features of this real-world intervention that we studied could serve to make it more or less effective. Ideally, future work should carefully curate other interventions which vary some intervention features (e.g., what tips and techniques are taught) while holding others constant (e.g., population tailoring, duration, multimodality) to best learn about what features of the intervention make it most effective. In addition to enriching our understanding of the mechanisms underlying the efficacy of digital media literacy interventions for older adults, work taking this approach can also serve to inform practitioners as they seek to scale these interventions as efficiently as possible and to deliver them to different audiences. In terms of modifying which digital literacy tips and techniques are taught in an intervention for older adults, our data suggest that older adults in our study had different baseline levels of comprehension for different digital literacy techniques and experienced different levels of improvement in that comprehension as a result of our intervention. These data provide initial evidence that digital literacy is not a monolith among older adults and that the returns to devoting time training on some digital literacy skills may differ from others.

Finally, our study does not provide much information about the durability of the effects of participation in *MediaWise for Seniors*. Individuals took one post-intervention survey, immediately following their completion of the intervention. Future work should make use of longitudinal research designs, administering multiple follow-up surveys at a variety of time intervals, to understand the extent to which the benefits of participating in interventions like *MediaWise for Seniors* endure after the intervention has ended. Existing research demonstrates that effects of misinformation interventions can (fail to) endure in ways that are important to capture, such as Basol et al. who found evidence of increased skepticism of both true and false news after a prebunking intervention but that the heightened skepticism of true news disappeared 1 week after the intervention while skepticism toward false news persisted^40^. Basol et al. demonstrates the utility of examining the durability of misinformation interventions, as their intervention initially appeared to decrease trust in true news but that decrease did not persist 1 week later, in which an outcome much closer to discriminant trust was present.

As older adults increasingly adopt social media and make use of modern communication technologies to consume news, it will be important to ensure that they have the skills necessary to sort true from false content and protect themselves from misinformation. Older adults turnout to vote at consistently higher rates than younger individuals in democracies around the globe^41,42^, so the consequences of their belief in political falsehoods could have meaningful electoral consequences. If older adults, a civic-minded and community-oriented demographic group’s, ability to identify misinformation online can be improved, there are reasons to believe that they could become a valuable resource for society at large in its fight against misinformation and other falsehoods which proliferate online^43^.

## Supplementary Information

Supplementary Information.

## References

[1] Grinberg N, Joseph K, Friedland L, Swire-Thompson B, Lazer D (2019). Fake news on Twitter during the 2016 U.S. presidential election. Science.

[2] Guess A, Nagler J, Tucker J (2019). Less than you think: Prevalence and predictors of fake news dissemination on Facebook. Sci. Adv..

[3] Allen J, Howland B, Mobius M, Rothschild D, Watts DJ (2020). Evaluating the fake news problem at the scale of the information ecosystem. Sci. Adv..

[4] Brashier NM, Schacter DL (2020). Aging in an era of fake news. Curr. Dir. Psychol. Sci..

[5] Kozyreva A, Lewandowsky S, Hertwig R (2020). Citizens versus the internet: Confronting digital challenges with cognitive tools. Psychol. Sci. Public Interest.

[6] Sparks JR, Katz IR, Beile PM (2016). Assessing digital information literacy in higher education: A review of existing frameworks and assessments with recommendations for next-generation assessment. ETS Res. Rep. Ser..

[7] Hargittai E, Piper AM, Morris MR (2019). From internet access to internet skills: Digital inequality among older adults. Univ. Access Inf. Soc..

[8] van Deursen A, van Dijk J (2010). Internet skills and the digital divide. New Media Soc..

[9] Hargittai E, Dobransky K (2017). Old dogs, new clicks: Digital inequality in skills and uses among older adults. Can. J. Commun..

[10] Friemel TN (2016). The digital divide has grown old: Determinants of a digital divide among seniors. New Media Soc..

[11] Berenguer A (2017). Are smartphones ubiquitous?: An in-depth survey of smartphone adoption by seniors. IEEE Consum. Electron. Mag..

[12] Span, P. Getting wise to fake news. *The New York Times* (2020).

[13] Wineburg, S. & McGrew, S. Lateral reading and the nature of expertise: reading less and learning more when evaluating digital information. *Teachers College Record* **121** (2019).

[14] Schaffel, G. Facebook most popular with older users. *AARP*. http://www.aarp.org/home-family/personal-technology/info-2018/facebook-users-age-fd.html (2018).

[15] Kahne J, Bowyer B (2017). Educating for democracy in a partisan age: Confronting the challenges of motivated reasoning and misinformation. Am. Educ. Res. J..

[16] McGrew S, Smith M, Breakstone J, Ortega T, Wineburg S (2019). Improving university students’ web savvy: An intervention study. Br. J. Educ. Psychol..

[17] Badrinathan S (2021). Educative interventions to combat misinformation: Evidence from a field experiment in India. Am. Polit. Sci. Rev..

[18] Hunsaker A, Hargittai E (2018). A review of Internet use among older adults. New Media Soc..

[19] Vaportzis E, Giatsi Clausen M, Gow AJ (2017). Older adults perceptions of technology and barriers to interacting with tablet computers: A focus group study. Front. Psychol..

[20] Levine TR (2019). Duped: Truth-Default Theory and the Social Science of Lying and Deception.

[21] Kim RK, Levine TR (2011). The effect of suspicion on deception detection accuracy: Optimal level or opposing effects?. Commun. Rep..

[22] McCornack SA, Levine TR (1990). When lovers become leery: The relationship between suspicion and accuracy in detecting deception. Commun. Monogr..

[23] Luo M, Hancock JT, Markowitz DM (2022). Credibility perceptions and detection accuracy of fake news headlines on social media: Effects of truth-bias and endorsement cues. Commun. Res..

[24] Edelman. *2021 Edelman Trust Barometer*. 58 https://www.edelman.com/trust/2021-trust-barometer (2021).

[25] Van Duyn E, Collier J (2019). Priming and fake news: The effects of elite discourse on evaluations of news media. Mass Commun. Soc..

[26] Guess AM (2020). A digital media literacy intervention increases discernment between mainstream and false news in the United States and India. PNAS.

[27] Guess A, Nyhan B, Reifler J (2020). Exposure to untrustworthy websites in the 2016 US election. Nat. Hum. Behav..

[28] Gringlas, S. With an election on the horizon, older adults get help spotting fake news. *NPR.org.* https://www.npr.org/2020/02/26/809224742/with-an-election-on-the-horizon-older-adults-get-help-spotting-fake-news (2020).

[29] Kujala S, Roto V, Väänänen-Vainio-Mattila K, Karapanos E, Sinnelä A (2011). UX Curve: A method for evaluating long-term user experience. Interact. Comput..

[30] Pearce KE, Rice RE (2013). Digital divides from access to activities: Comparing mobile and personal computer internet users. J. Commun..

[31] Tsai HS, Shillair R, Cotten SR, Winstead V, Yost E (2015). Getting grandma online: Are tablets the answer for increasing digital inclusion for older adults in the U.S.?. Educ. Gerontol..

[32] Cao J, Shu K, Wang S, Lee D, Liu H (2020). Exploring the role of visual content in fake news detection. Disinformation, Misinformation, and Fake News in Social Media: Emerging Research Challenges and Opportunities.

[33] Roozenbeek J, Maertens R, McClanahan W, van der Linden S (2021). Disentangling item and testing effects in inoculation research on online misinformation: Solomon revisited. Educ. Psychol. Meas..

[34] Maruf, R. These four words are helping spread vaccine misinformation. *CNN.* https://www.cnn.com/2021/09/19/media/reliable-sources-covid-research/index.html (2021).

[35] Hargittai E, Hsieh YP (2011). Succinct survey measures of web-use skills. Soc. Sci. Comput. Rev..

[36] Levine TR, Shaw AS, Shulman H (2010). Assessing deception detection accuracy with dichotomous truth-lie judgments and continuous scaling: Are people really more accurate when honesty is scaled?. Commun. Res. Rep..

[37] R Core Team (2021). R: A Language and Environment for Statistical Computing.

[38] StataCorp (2021). Stata Statistical Software: Release 17.

[39] Silverman, C. Old, online, and fed on lies: how an aging population will reshape the Internet. *BuzzFeed News*. https://www.buzzfeednews.com/article/craigsilverman/old-and-online-fake-news-aging-population (2019).

[40] Basol M (2021). Towards psychological herd immunity: Cross-cultural evidence for two prebunking interventions against COVID-19 misinformation. Big Data Soc..

[41] Bunis, D. The immense power of the older voter in an election. *AARP*. http://www.aarp.org/politics-society/government-elections/info-2018/power-role-older-voters.html (2018).

[42] Franklin MN (2004). Voter Turnout and the Dynamics of Electoral Competition in Established Democracies since 1945.

[43] Moore RC, Hancock JT (2020). Older adults, social technologies, and the coronavirus pandemic: Challenges, strengths, and strategies for support. Soc. Media Soc..

