# Supplementary material for: A digital media literacy intervention for older adults improves resilience to fake news
Source: Sci Rep. 2022 Apr 9;12:6008. doi: 10.1038/s41598-022-08437-0 (PMC8994776; doi:10.1038/s41598-022-08437-0)
Supplement: Supplementary file 1 — Supplementary Information. [file 41598_2022_8437_MOESM1_ESM.docx]

Supplementary Materials for

**A Digital Media Literacy Intervention for Older Adults Improves Resilience to Fake News**

**Digital Literacy Concepts Taught in MediaWise for Seniors**

**Lateral reading** refers to the principle of opening new web browser tabs and conducting searches and reading across multiple tabs to see what other sources say about a claim. **Click restraint** refers to search engine results and involves resisting the urge to immediately click on the first search result, instead scanning the results to make a more informed choice about where to go first. **Reverse image search** refers to conducting a search of an image to identify if and which other webpages have published that same image. **Wikipedia page features** refer to the references, edit histories, annotations, and other attributes that provide context and greater detail for a Wikipedia entry. **Search engine query optimization** refers to the use of boolean operators, date windows, and other advanced search queries to conduct a search that is more likely to surface results relevant to one’s query. Finally, **search engine filters** refer to built-in category filters that one can use to only view certain types of results for their query (e.g., news, videos).

**Results by Congeniality of Headlines**

One question to answer when evaluating interventions aimed at improving individuals’ ability to identify misinformation is whether an intervention is similarly effective for both misinformation that is congenial and not congenial with their prior beliefs (Kahne & Bowyer, 2017). Given the partisan nature of the headlines participants were tested on in our surveys (see Table S1), we can examine whether the accuracy-enhancing effects of *MediaWise for Seniors* differ for politically congenial (a Democrat judging a Democrat-congenial headline or a Republican judging a Republican-congenial headline) and non-congenial headlines (a Democrat judging a Republican-congenial headline or a Republican judging a Democrat-congenial headline).^^[[1]](#footnote-1)^^ The percentage of veracity judgments that were accurate by condition (treatment, control), survey wave (pre-intervention survey, post-intervention survey) and congeniality (congenial headlines, non-congenial headlines) are presented in Table S7. The differences in pre-post change in the proportion of congenial headlines accurately detected between the treatment and control group was 25% while for non-congenial headlines the difference in pre-post change was 29%. Descriptively, this suggests that the intervention seemed to improve the treatment group’s accuracy for non-congenial headlines more than for congenial headlines, although the difference is small (4%). However, due to our low sample size, we cannot confidently make statistical claims about this effect. Future work which recruits sufficient sample sizes to detect this three-way interaction between condition, time, and congeniality should explore this aspect of intervention efficacy further.

*Table S1: News headlines used in deception detection task*

|  | True News | False News |
| --- | --- | --- |
| Republican congenial | **Donald Trump Sends $10,000 to Hero Bus Driver after Driver Saves Suicidal Woman from Jumping off Bridge**  Bus driver Darnell Barton took a detour from his normal route in order to stop a woman from jumping off an overpass. Trump said he was moved by the gesture and rewarded Barton with a $10,000 check.  *Pre-intervention survey* | **Joe Biden Waves at Empty Field as He Departs Plane in Tampa**  The 77-year-old was caught waving to an empty field after departing a plane in Tampa on Tuesday. He was in Tampa holding a veterans roundtable.  *Pre-intervention survey* |
|  | **Child with Rare Ailment Rescued Aboard Trump's Private Jet**  Donald Trump's private jet carried a critically ill 3-year-old Jewish boy from California to New York for medical treatment after commercial airlines refused to carry the boy.  *Post-intervention survey* | **Biden Caught Using Teleprompter During Interview**  Democratic presidential nominee Joe Biden is caught red-handed using a teleprompter during a recent interview with Telemundo anchor Jose Diaz-Balart.  *Post-intervention survey* |
| Democrat congenial | **Trump Administration Secretly Withheld Millions from FDNY 9/11 Health Program**  The Trump administration has secretly siphoned nearly $4 million away from a program that tracks and treats FDNY firefighters and medics suffering from 9/11 related illnesses.  *Pre-intervention survey* | **Semi Truck Bearing the Words "All Aboard the Trump Train" Crashes Into Overpass**  The truck wedged itself under the Mamaroneck Avenue Overpass in New York on Monday.  *Pre-intervention survey* |
|  | **Republican Candidate Made Unbelievable Statement About Rape**  Clayton Williams, former Republican candidate for Texas Governor, likened rape to bad weather, stating, "Rape is kinda like the weather. If it's inevitable, relax and enjoy it." Williams made the remark to reporters at his ranch in West Texas.  *Post-intervention survey* | **Trump Refuses to Send Wildfire Aid to CA, Offers it to Putin**  Donald Trump has refused to provide federal assistance to California to help them fight against September 2020 wildfires. In July, however, Trump offered US aid to Russian President Vladimir Putin to help fight against fires that broke out in Russia.  *Post-intervention survey* |
| Neither Republican nor Democrat congenial | **Netflix Releases Film Poster Portraying Young Girls in Sexualized Manner**  The movie, titled "Mignonnes", follows an 11-year-old Senegalese Muslim girl who is caught up in the clash of her traditional family values and modern, internet culture.  *Pre-intervention survey* | **Dozens of Countries Ordered COVID-19 Tests in 2018** Data displayed on the World Bank's World Integrated Trade Solution website in September 2020 proved that dozens of nations imported or exported COVID-19 Test Kits in 2018 — more than a year before the pandemic occurred.  *Pre-intervention survey* |
|  | **Walmart Customer’s Change Donated Without Consent**  In July, a Walmart customer in Massachusetts had the change from her purchase donated to charity without her consent. The customer says a Walmart cashier refused to give her change with no advanced warning.  *Post-intervention survey* | **Adolf Hitler Found to Have Invented the Inflatable Sex Doll**  Hitler's Borghild Project, which began in late 1940, was a secretive attempt to stop the spread of syphilis by providing Nazi soldiers with inflatable sex dolls.  *Post-intervention survey* |

*Table S2: Correctly judging news headline veracity*

|  | All Headlines | True Headlines | False Headlines |
| --- | --- | --- | --- |
| Intervention Group (1=Intervention group,  0=Control group) | 0.348***  (0.086) | 0.278*  (0.127) | 0.455**  (0.139) |
| Post-intervention (1=Post intervention survey,  0=Pre survey) | 0.080  (0.070) | 0.303**  (0.109) | -0.162  (0.102) |
| Intervention Group * Post-intervention | 1.073***  (0.159) | 1.570***  (0.265) | 0.580**  (0.200) |
| Observations | 3,838 | 1,897 | 1,941 |
| AIC | 4,841 | 2,390 | 2,360 |

*Note*: “Intervention Group” variable: control group = 0; intervention group = 1. “Post-intervention” variable: post-intervention survey = 1; pre-intervention survey = 0. Dependent variable in “All Headlines” model: an accurate headline veracity judgment = 1; an inaccurate headline veracity judgment = 0. Dependent variable in “True Headlines” model: a headline veracity judgment of true = 1; a headline veracity judgment of false = 0. Dependent variable in “False Headlines” model: a headline veracity judgment of false = 1; a headline veracity judgment of true = 0. Regression coefficients are shown with standard errors (clustered on participants) in parentheses. *p < .05, **p < .01, ***p < .001.

*Table S3: Correctly judging news headline veracity (non-dichotomized veracity judgments)*

|  | All Headlines | True Headlines | False Headlines |
| --- | --- | --- | --- |
| Intervention Group (1=Intervention group,  0=Control group) | 0.332***  (0.077) | 0.313**  (0.115) | 0.353***  (0.103) |
| Post-intervention (1=Post intervention survey,  0=Pre survey) | 0.077  (0.070) | 0.314**  (0.105) | -0.161  (0.103) |
| Intervention Group * Post-intervention | 1.202***  (0.130) | 1.620***  (0.184) | 0.782***  (0.157) |
| Observations | 4,574 | 2,288 | 2,286 |
| R^2^ | 0.072 | 0.130 | 0.037 |

*Note*: “Intervention Group” variable: control group = 0; intervention group = 1. “Post-intervention” variable: post-intervention survey = 1; pre-intervention survey = 0. Dependent variable in “All Headlines” model: the raw veracity judgment given for a headline, ranging from 1 (definitely false) - 7 (definitely true). For false headlines, this judgment was reverse coded such that higher values represent more accurate judgments. Dependent variable in “True Headlines” model: the raw veracity judgment given for a headline, ranging from 1-7. Dependent variable in “False Headlines” model: the raw veracity judgment given for a headline, ranging from 1-7, reverse coded such that higher values represent more accurate judgements. Regression coefficients are shown with standard errors (clustered on participants) in parentheses. **p < .01, ***p < .001.

*Table S4: Use and comprehension of digital literacy skills*

|  | Researching |  | Lateral reading | Click restraint | Reverse image search | Wikipedia page features | Search engine query optimization | Search engine filters |
| --- | --- | --- | --- | --- | --- | --- | --- | --- |
|  | *logistic* |  | *OLS* | | | | | |
| Intervention Group (1=Intervention group,  0=Control group) | 0.397  (0.437) |  | 0.140  (0.110) | 0.175  (0.112) | 0.105  (0.136) | 0.109  (0.133) | 0.364**  (0.134) | 0.025  (0.134) |
| Post-intervention (1=Post intervention survey,  0=Pre survey) | -0.330  (0.338) |  | -0.117  (0.066) | -0.080  (0.060) | -0.090  (0.067) | -0.003  (0.081) | -0.142  (0.082) | -0.135  (0.074) |
| Intervention Group * Post-intervention | 4.436***  (0.505) |  | 2.722***  (0.128) | 2.741**  (0.132) | 1.941***  (0.135) | 1.587***  (0.133) | 1.247***  (0.137) | 1.027***  (0.128) |
| Observations | 4,562 |  | 761 | 761 | 761 | 760 | 761 | 761 |
| R^2^ |  |  | 0.545 | 0.546 | 0.277 | 0.204 | 0.170 | 0.082 |
| AIC | 1,898 |  |  |  |  |  |  |  |

*Note:* “Intervention Group” variable: control group = 0; intervention group = 1. “Post-intervention” variable: post-intervention survey = 1; pre-intervention survey = 0. Dependent variable in “Researching” model: a participant reported doing research on a headline to inform their judgment of its veracity = 1; a participant reported *not* doing research on a headline = 0. Dependent variable in “Lateral reading” model: a participant’s reported level of understanding of Lateral reading from 1-5 where a rating of 1 represents no understanding and a rating of 5 represents full understanding. Dependent variable in “Click restraint” model: a participant’s reported level of understanding of Click restraint from 1-5. Dependent variable in “Reverse image search” model: a participant’s reported level of understanding of Click restraint from 1-5. Dependent variable in “Wikipedia page features” model: a participant’s reported level of understanding of Wikipedia page features from 1-5. Dependent variable in “Search engine query optimization” model: a participant’s reported level of understanding of Search engine query optimization from 1-5. Dependent variable in “Search engine filters” model: a participant’s reported level of understanding of Search engine filters from 1-5. “Researching” model is a logistic regression model and all others use OLS. Regression coefficients are shown with standard errors (clustered on participants) in parentheses. **p < .01, ***p < .001.

*Table S5: Correctly judging news headline veracity among intervention group and control group respondents who indicated interest in participating in intervention*

|  | All Headlines | True Headlines | False Headlines |
| --- | --- | --- | --- |
| Intervention Group (1=Intervention group,  0=Control group) | 0.013  (0.135) | -0.043  (0.183) | 0.106  (0.222) |
| Post-intervention (1=Post intervention survey,  0=Pre survey) | 0.252  (0.142) | 0.590**  (0.213) | -0.072  (0.233) |
| Intervention Group * Post-intervention | 1.242***  (0.209) | 1.631***  (0.332) | 0.884**  (0.306) |
| Observations | 1,417 | 715 | 702 |
| AIC | 1,767 | 841 | 908 |

*Note*: “Intervention Group” variable: control group members who indicated interest in participating in MediaWise for Seniors upon being shown marketing material for the course = 0; intervention group = 1. “Post-intervention” variable: post-intervention survey = 1; pre-intervention survey = 0. Dependent variable in “All Headlines” model: an accurate headline veracity judgment = 1; an inaccurate headline veracity judgment = 0. Dependent variable in “True Headlines” model: a headline veracity judgment of true = 1; a headline veracity judgment of false = 0. Dependent variable in “False Headlines” model: a headline veracity judgment of false = 1; a headline veracity judgment of true = 0. Regression coefficients are shown with standard errors (clustered on participants) in parentheses. **p < .01, ***p < .001.

*Table S6: Use and comprehension of digital literacy skills among intervention group and control group respondents who indicated interest in participating in intervention*

|  | Researching |  | Lateral reading | Click restraint | Reverse image search | Wikipedia page features | Search engine query optimization | Search engine filters |
| --- | --- | --- | --- | --- | --- | --- | --- | --- |
|  | *logistic* |  | *OLS* | | | | | |
| Intervention Group (1=Intervention group,  0=Control group) | 0.637  (0.564) |  | 0.024  (0.148) | -0.0002  (0.156) | 0.011  (0.186) | -0.112  (0.186) | -0.005  (0.186) | -0.067  (0.182) |
| Post-intervention (1=Post intervention survey,  0=Pre survey) | 0.583**  (0.198) |  | -0.181  (0.117) | -0.093  (0.119) | -0.052  (0.108) | -0.007  (0.140) | -0.245  (0.143) | -0.087  (0.126) |
| Intervention Group * Post-intervention | 3.523***  (0.425) |  | 2.786***  (0.160) | 2.753***  (0.167) | 1.902***  (0.159) | 1.591***  (0.176) | 1.351***  (0.181) | 0.979***  (0.164) |
| Observations | 2,642 |  | 442 | 441 | 439 | 442 | 441 | 441 |
| R^2^ |  |  | 0.621 | 0.592 | 0.338 | 0.255 | 0.171 | 0.108 |
| AIC | 1,521 |  |  |  |  |  |  |  |

*Note:* “Intervention Group” variable: control group members who indicated interest in participating in MediaWise for Seniors upon being shown marketing material for the course = 0; intervention group = 1. “Post-intervention” variable: post-intervention survey = 1; pre-intervention survey = 0. Dependent variable in “Researching” model: a participant reported doing research on a headline to inform their judgment of its veracity = 1; a participant reported *not* doing research on a headline = 0. Dependent variable in “Lateral reading” model: a participant’s reported level of understanding of Lateral reading from 1-5 where a rating of 1 represents no understanding and a rating of 5 represents full understanding. Dependent variable in “Click restraint” model: a participant’s reported level of understanding of Click restraint from 1-5. Dependent variable in “Reverse image search” model: a participant’s reported level of understanding of Click restraint from 1-5. Dependent variable in “Wikipedia page features” model: a participant’s reported level of understanding of Wikipedia page features from 1-5. Dependent variable in “Search engine query optimization” model: a participant’s reported level of understanding of Search engine query optimization from 1-5. Dependent variable in “Search engine filters” model: a participant’s reported level of understanding of Search engine filters from 1-5. “Researching” model is a logistic regression model and all others use OLS. Regression coefficients are shown with standard errors (clustered on participants) in parentheses. **p < .01, ***p < .001.

*Table S7: Accuracy of headline veracity judgments by condition, survey wave, and headline congeniality*

| **Condition** | **Survey wave** | **Congeniality of headlines** | **Percentage of judgments that were accurate** | **Pre to post change** | **Difference in pre to post change between conditions** |
| --- | --- | --- | --- | --- | --- |
| Treatment | Pre | Non-congenial | 45% | +42% | 29% |
| Treatment | Post | Non-congenial | 87% |  |  |
| Control | Pre | Non-congenial | 43% | +13% |  |
| Control | Post | Non-congenial | 56% |  |  |
| Treatment | Pre | Congenial | 55% | +21% | 25% |
| Treatment | Post | Congenial | 76% |  |  |
| Control | Pre | Congenial | 51% | -4% |  |
| Control | Post | Congenial | 47% |  |  |

*Figure S1: Proportion of accurate veracity judgments by headline*


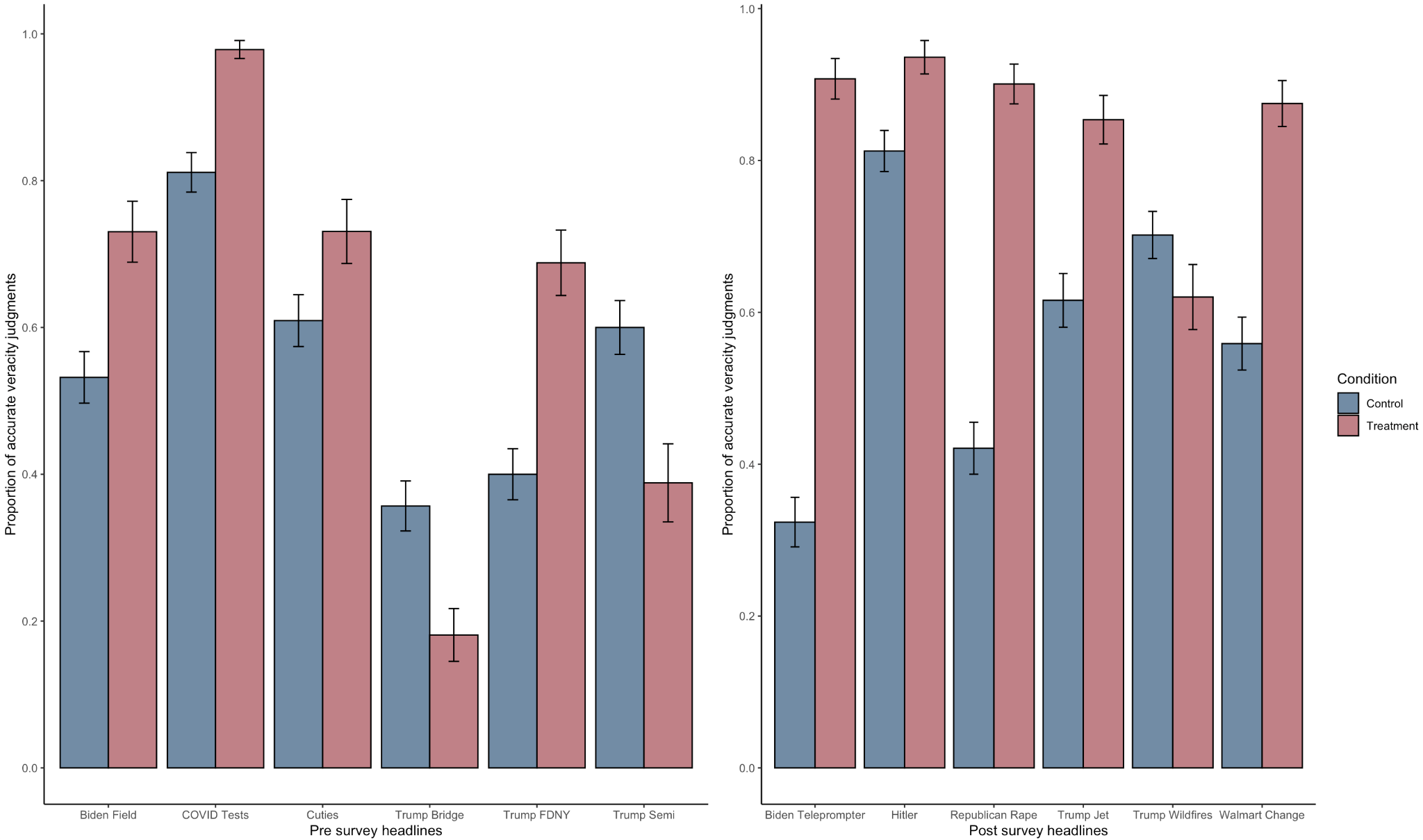


*Note:* Proportion of accurate veracity judgments by headline. The left graph includes the six headlines contained in the pre-intervention survey and the right graph includes the six headlines contained in the post-intervention survey (see Table S1). The red bars represent the treatment group and the blue bars represent the control group. Error bars are standard errors.

1. Respondents self-reported their partisan identification, those who reported identifying as Independents were classified as whichever party they reported their beliefs being closer to, Democratic or Republican. [↑](#footnote-ref-1)
